# Overcoming salinity barriers: granular straw promotes carbon cycling in saline soils by reshaping microbial community assembly

**DOI:** 10.3389/fmicb.2026.1782607

**Published:** 2026-04-29

**Authors:** Ting Fan, Kexin Hu, Shaoqi Xue, Pengfei Li, Yulin Zhang, Xudong Wang

**Affiliations:** 1 Yinshanbeilu Grassland Eco-Hydrology National Observation and Research Station, China Institute of Water Resources and Hydropower Research, Beijing, China; 2 Institute of Pastoral Hydraulic Research, Hohhot, Inner Mongolia, China; 3 College of Resources and Environment, Northwest A&F University, Yangling, Shaanxi, China; 4 Key Laboratory of Plant Nutrition and Agri-environment in Northwest China, Ministry of Agriculture, Yangling, Shaanxi, China

**Keywords:** biochar, granular straw, soil microorganism, soil salinity, temperature

## Abstract

Saline soils, characterized by elevated salinity, depleted soil organic carbon (SOC), and degraded structure, constrain global agricultural productivity. While straw incorporation is known to promote SOC turnover, its efficacy in saline soils is limited by salt-impaired microbial decomposition. We evaluated how straw form regulates microbial-driven SOC turnover across salinity gradients (light-S1, moderate-S2, severe-S3) and temperatures (10 °C-T1, 20 °C-T2, 30 °C-T3) through a 360-day incubation. Treatments included conventional chopped wheat straw (CW), granular wheat straw (GW), wheat straw biochar (BW), and no-straw control (CK). Straw form significantly altered fungal α-diversity (*p* < 0.05) but not bacterial. Fungal diversity was enhanced by BW and T3, while S2 exhibited maximal fungal richness. *Ascomycota* dominated fungal communities (65.0–97.7%), with GW increasing their relative abundance. Higher temperatures reduced *Ascomycota* dominance (T1 > T2 > T3, *p* < 0.05). Multivariate analyses confirmed that straw form, temperature, and salinity collectively drove distinct clustering of microbial communities. Under the same environmental temperature and soil salinity, the soil fungal communities of each treatment were significantly separated according to the different forms of straw application. Among them, the distribution of soil fungal communities in the GW treatment differed significantly from other treatments. Co-occurrence networks revealed GW enhanced microbial network complexity and stability. The Mantel test analysis showed that straw form to SOC turnover via microbial restructuring, demonstrating stronger fungal-physicochemical correlations than bacterial. Critically, GW altered microbial community structure, as evidenced by the distinct separation of fungal communities and increased complexity of co-occurrence networks, and promoted SOC turnover. These findings establish granular straw as an effective practice to overcome the decomposition barriers inherent to conventional straw in saline soils, thereby promoting nutrient cycling and supporting sustainable land management.

## 1 Introduction

Secondary salinization has been considered as a serious factor for the degradation of many fertile arable soils worldwide, and thus constraining soil fertility and threatening global ecological balance. China faces significant challenges due to extensive saline-alkali soils, with approximately 20% of arable land affected by salinization. The Hexi Corridor in Gansu Province, a representative saline-alkali region in Northwest China, encompasses 16,100 hm^2^ of saline-alkali land. This region is also characterized by its cold, low-temperature climate with significant temperature fluctuations, especially during the critical periods for straw incorporation and decomposition. Soil salinity critically regulates microbial abundance and diversity. Excessive soil salinity induces microbial inactivation and reduces population density (El-Akhdar et al., 2025). Wang Z. et al. (2025) observed salinity-driven shifts in microbial community structure. Bacterial and fungal taxa exhibit distinct salt tolerance thresholds. Zhang S. et al. (2021) indicated that fungal community shows higher tolerance, stability, and resilience to various saline-alkaline soils than a bacterial community through the correlation network analysis. Research has also shown that soil microorganisms, namely *Nocardioides*, *Saccharimonadaceae*, and *Nitriliruptoraceae* bacteria, can adapt to high-salinity environments, thereby accelerating nutrient cycling in saline soils (Chen et al., 2025).

Straw incorporation has emerged as a critical strategy for ameliorating saline soils. Exogenous straw application not only modulates soil physicochemical properties and fertility but also reshapes microbial habitats and community composition. However, crop straw is usually spread or overturned into topsoil after harvest, and adverse effects have been detected during the agricultural activity and growth of the subsequent crop, especially in arid and semiarid areas. For example, crop residues can block the sowing machine, cause soil towage, and even inhibit the emergence and growth of seeds (Wang et al., 2020). Biochar has a rich pore structure and large specific surface area and contains abundant stable carbon elements. It has good application potential for improving the physical and chemical properties of soils and farmland, reducing greenhouse gas emissions and increasing the “agricultural carbon sink” (Fang et al., 2015). However, few studies have investigated the impact of straw biochar on microbial communities in different saline soils. In recent years, the application of condensed organic materials of straw (granular straw) to agricultural fields has been reported (Cong et al., 2020b). High-density granular straw made of crop straw not only prevents clogging during the operation of agricultural machinery but also minimizes difficulties associated with straw accommodation in the soil during short fallow periods. However, the changes in soil microbial community structure following the addition of different forms of straw in soils with varying degrees of salinization remain to be studied.

Soil microorganisms play a pivotal role in driving nutrient cycling and maintaining global carbon balance. The turnover of the soil organic carbon (SOC) pool is a synergistic outcome of microbial interactions between soil bacteria and fungi (Wu et al., 2025). Research indicates that straw return practices significantly alter soil microbial community structure, as the carbon and nitrogen in straw provide essential substrates and energy for microbial proliferation (Wu et al., 2024). Yang et al. (2022) demonstrated that wheat straw incorporation markedly increases the abundance of soil bacteria and fungi. Similarly, Wu et al. (2023) reported that straw incorporation increased bacterial diversity by 3.6% and microbial functional genes abundances of C, N and P cycling under warming soil conditions. However, contrasting studies reveal that biochar application in sandy soils with low organic carbon content reduces microbial biomass (Li et al., 2020). These results suggest that, in addition to soil type and fertility factors, different forms of homologous straw may exert divergent effects on microbial communities. Such differences are potentially attributable to alterations in physical structure, chemical composition, and bioavailability resulting from changes in straw morphology. Therefore, in-depth investigation into the effects of different straw forms on microbial communities is crucial for precisely understanding soil organic carbon turnover mechanisms and elucidating microbially driven carbon dynamics.

Temperature is a critical external factor shaping soil microbial community structure. Fluctuations in temperature alter microbial metabolic activity and proliferation rates, thereby modifying community composition (Zhang et al., 2022). Frossard et al. (2021) demonstrated that soil microbial growth and activity peak at 25–35 °C, with higher temperatures within this range correlating with increased microbial abundance and metabolic vigor. Dai et al. (2020) observed that elevated temperatures intensify microbial degradation of organic residues. However, Hopkins et al. (2014) reported that warming increases microbial biomass without altering taxonomic composition. Divergent responses between bacterial and fungal communities to temperature shifts have also been documented. For instance, Ma et al. (2022) found that soil temperature were strongly associated with bacterial diversity, but less well correlated with fungal diversity. These differential responses may stem from variations in microbial physiological traits and nutrient acquisition strategies. Clarifying temperature adaptation mechanisms of soil bacteria and fungi under diverse conditions is vital for enhancing soil carbon sequestration and global carbon balance.

Currently, most studies are limited to the comparison of several different types of organic materials. There is relatively little research on the effects of different forms of straw from the same source, under equal carbon input, on soil microbial community structures across varying levels of salinity. Moreover, the application of high amounts of traditional chopping straw to improve saline soils often affects seeding and seedling emergence. Therefore, compressing straw into granular straw or making biochar and then returning it to the field may be better options to improve saline soils. However, at present, the succession patterns of microbial community structure after different forms of straw are mixed into saline soils have not been reported. Building on existing insights, in this study, we hypothesize that granular straw, compared to chopped straw, may further optimize microbial community structures. We propose that this optimization is particularly pronounced in the cold, low-temperature, and saline-alkali soils typical of Northwest China. To test this hypothesis under environmentally relevant conditions, a controlled incubation experiment was conducted by incorporating iso-carbon doses of homologous wheat straw in three forms—chopped straw, straw-derived biochar, and granular straw—into saline soils of varying salinity levels under different temperature regimes. The objectives were to (i) evaluate the impact of wheat straw forms on microbial community structure in saline-alkali soils and (ii) Determine the impact of microbial changes on soil organic carbon mineralization and propose optimal straw incorporation practices. This study provides novel insights into the biological mechanisms governing saline soil remediation under diverse straw management strategies and advances the feasibility of granular straw as a sustainable amendment for saline-alkali land restoration.

## 2 Materials and methods

### 2.1 Study site and soil characteristics

The experimental soil was collected from Gaotai County (39 °59 ′52 ″ N, 100 °06 ′42 ″ E), situated within the Hexi Corridor Irrigation District of Gansu Province, Northwest China. This region lies in the central Hexi Corridor and the lower-middle reaches of the Heihe River Basin. According to the World Reference Base for Soil Resources (WRB; ISSS/ISRIC/FAO, 1998), the soil is classified as Gypsisols, reflecting its dominant gypsum-rich profile. The area experiences a hyperarid continental desert climate, characterized by extreme temperature fluctuations, limited precipitation (mean annual: 103 mm), and exceptionally high evaporation (≥2,000 mm). The average annual temperature is 7.4 °C, with a frost-free period of approximately 150 days. Notably, saline-alkali soils in Gaotai County cover 16,100 hm^2^, constituting 64.5% of the local arable land-a critical indicator of the severity of soil degradation in this agroecologically vulnerable region.

### 2.2 Soil sampling and pretreatment

Soil samples were collected in June 2022 from the 0–20 cm plow layer of a conventionally managed wheat-cropped field in Gaotai County, Gansu Province. Following harvest of the preceding wheat crop, visible plant residues (roots, stems, and leaves) and macrofauna were manually removed to minimize organic matter heterogeneity. Fresh subsamples were immediately stored at −4 °C for microbial analyses, while the remaining soil was air-dried, homogenized, and sieved (<2 mm) for subsequent incubation experiments. Basic physicochemical properties of the soil (Table 1) were determined using standardized protocols (Pansu and Gautheyrou, 2006). Soil organic carbon (SOC): Potassium dichromate oxidation-external heating method. Total nitrogen (TN): Kjeldahl digestion–distillation method. Alkali-hydrolyzable nitrogen (AN): Alkaline diffusion method. Phosphorus fractions: Total phosphorus (TP) and available phosphorus (AP) quantified via molybdenum–antimony anti-colorimetry. Available potassium (AK): Flame photometry after ammonium acetate extraction. Particle size distribution: Pipette method with sodium hexametaphosphate dispersion. Total soluble salts: Gravimetric titration of saturation extract.

**Table 1:** Basic characteristics of various forms of wheat straw.

Straw form	pH	Bulk density (g cm^−3^)	Nutrient content			
			C %	N %	P %	K %
Chopped straw (CW)	7.2	0.19	47.5	1.9	0.1	1.7
Biochar straw (BW)	8.8	0.21	57.6	2.3	0.1	5.4
Granular straw (GW)	7.3	1.06	49.6	2.0	0.1	1.6

### 2.3 Soil salinity adjustment

The initial soil exhibited a salinity content of 2.52 g kg^−1^ (S1), classified as mildly saline based on established thresholds for arid agricultural soils (Shahid and Rehman, 2011). To simulate varying salinity gradients, air-dried soil (200 g dry weight) was transferred into 500 mL culture bottles and pre-incubated with 30 mL deionized water at 25 °C for 7 days to equilibrate moisture. Salinity was then systematically elevated by adding a synthetic salt solution formulated to mirror the base cation composition (Na^+^: Ca^2+^: Mg^2+^: K^+^ = 4.3: 2.1: 1.8: 1) of the native soil. The solution contained NaHCO₃ (8.8%), MgSO₄ (46.9%), CaCl₂ (13.5%), KCl (2.8%), NaCl (7.2%), and Na₂SO₄ (20.8%) (w/w). Incremental additions generated two salinity regimes: 4.89 g kg^−1^ (S2, moderately saline) and 7.25 g kg^−1^ (S3, severely saline). The two elevated salinity levels (S2 and S3) were selected to represent the range of salinities commonly observed in moderately to severely saline fields within the Hexi Corridor region, based on a survey of local soil conditions. Post-adjustment, soils were re-incubated at 25 °C for 7 days under dark conditions to stabilize ionic distributions and minimize microbial activity artifacts.

### 2.4 Straw forms

#### 2.4.1 Feedstock processing

Wheat straw was air-dried, partially crushed into 1–2 cm fragments, and stored in sealed plastic bags. A portion of the straw was used to produce granulated straw, while the remainder was converted to biochar.

#### 2.4.2 Biochar preparation

Chopped straw was subjected to oxygen-limited pyrolysis in a muffle furnace (Yamato FO410C) preheated to 350 °C. To ensure anoxic conditions, samples were sealed in stainless steel containers under continuous N₂ purging (1.5 L min^−1^) for 180 min. Following pyrolysis, the biochar was cooled to ambient temperature under sustained N₂ flow, homogenized, and stored in gas-tight bags to prevent hygroscopic degradation.

#### 2.4.3 Straw granulation

Dried was pulverized (<2 mm particle size) and mixed with a urea solution to standardize the C/N ratio to 25: 1. The mixture was then extruded into cylindrical granules (8 mm diameter × 1–2 cm length) using a ring-die granulators (SKJ-650, Shandong Zhangqiu Mingchuang Machinery Co., Ltd., China). All straw amendments-chopped, granulated, and biochar-were C/N-adjusted to 25: 1 to isolate physical form effects from stoichiometric variability. Key physicochemical properties of the amendments are detailed in Table 1. Total carbon (C) was determined via the potassium dichromate oxidation method; nitrogen (N), phosphorus (P), and potassium (K) concentrations in wheat tissues were quantified using the semi-micro Kjeldahl method, vanadate-molybdate yellow colorimetry, and flame photometry, respectively (Westerman, 1991).

### 2.5 Experimental design and methods

A controlled laboratory incubation study was conducted to investigate the effects of straw form on microbial community structure in saline-alkali soils, employing a three-factor full factorial design: Straw amendments (CK: no addition; BW: wheat-derived biochar; CW: chopped straw [1–2 cm]; GW: granular straw [8 mm diameter × 1–2 cm length]); Environment temperature, with three levels reflecting the seasonal extremes of the study region: T1 (10 °C, representing early spring / late autumn), T2 (20 °C, representing the growing season optimum), and T3 (30 °C, representing summer heat peaks); Soil salinity levels (S1: mild [2.52 g kg^−1^]; S2: moderate [4.89 g kg^−1^]; S3: severe [7.25 g kg^−1^]). This generated 36 treatments (4 amendments × 3 temperatures × 3 salinities), each with three replicates (n = 108 microcosms).

Air-dried saline soils (200 g dry weight equivalent per treatment) were thoroughly homogenized with iso-carbon equivalents of straw amendments. Chopped straw was applied at 12 g kg^−1^ soil (fresh weight basis), while biochar and granular straw were added at iso-carbon-adjusted rates based on their stoichiometric carbon contents (see Table 2 for stoichiometric details). Soil-amendment mixtures were packed into 500 mL borosilicate glass jars at a bulk density of 1.35 g cm^−3^, simulating *in situ* field conditions. Following a 72-h pre-incubation at 25 °C (75% field capacity) to equilibrate microbial activity post-moisture adjustment, microcosms were transferred to temperature-controlled incubators under dark conditions. Soil moisture was maintained gravimetrically at 75% field capacity by replenishing evaporated water with sterile deionized H₂O every 72 h throughout the 360-day incubation period.

**Table 2:** Alpha diversity indices of soil bacterial and fungi under different treatments.

Experimental factor	Experimental level	Bacteria			Fungi		
		Chao 1	Shannon	Simpson	Chao 1	Shannon	Simpson
Soil salinity (S)	Mild (S1)	2018 a	9.91 a	0.997 a	304.4 b	4.78 b	0.89 ab
	Moderate (S2)	2019 a	9.89 a	0.997 a	319.8 a	4.95 a	0.90 a
	Severe (S3)	1966 a	9.83 a	0.998 a	315.4 ab	4.77 b	0.88 b
Environment temperature (T)	Low (T1)	1998 b	9.91 b	0.997 ab	277.3 c	4.74 b	0.89 a
	Moderate (T2)	2,104 a	10.05 a	0.998 a	312.5 b	4.92 a	0.89 a
	High (T3)	1901 c	9.68 c	0.997 b	349.9 a	4.84 ab	0.89a
Straw forms(F)	No straw addition (CK)	1944 a	9.79 b	0.997 a	281.2 c	4.26 c	0.84 c
	Chopped straw (CW)	2040 a	9.96 a	0.998 a	270.6 c	4.42 c	0.87 b
	Biochar (BW)	1982 a	9.85 ab	0.997 a	358.8 a	5.43 a	0.94 a
	Granular straw (GW)	2037 a	9.92 ab	0.997 a	342.3 b	5.23 b	0.92 a
Significance (P)	S	0.438	0.321	0.336	0.084	0.053	0.024*
	T	<0.001**	<0.001**	0.049*	<0.001**	0.110	0.776
	F	0.231	0.049*	0.505	<0.001**	<0.001**	<0.001**
	S × T	0.142	0.205	0.451	0.001**	0.001**	0.01**
	S × F	0.041*	0.011*	0.154	0.003**	0.001**	0.006**
	T × F	0.092	0.054	0.350	<0.001**	<0.001**	<0.001**
	S × T × F	0.525	0.269	0.695	<0.001**	<0.001**	<0.001**

### 2.6 Sample analysis and data processing

After 360 days of incubation, the soil organic carbon mineralization (Ccum) was measured using gas chromatography (Agilent 7890B, Agilent Technologies, Santa Clara, CA, United States). The SOC was determined by the potassium dichromate oxidation method after removing carbonates with 1 mol L^−1^ HCl. The dissolved organic carbon (DOC) was measured by extracting soil with deionized water at a 1:10 soil: water ratio for 30 min at 250 rpm, and then centrifuging for 10 min at 8000 rpm; the supernatant solution was filtered with a 0.45 μm filter film for determining the DOC with a TOC-VCPH analyzer (Shimadzu Corp., Kyoto, Japan). The microbial biomass carbon (MBC) was measured with chloroform fumigation; the extract solution was determined by TOC analyzer -VCPH analyzer (Shimadzu Corp., Kyoto, Japan). The readily oxidizable organic carbon (ROC) was measured with 333 mol L^−1^ KMnO_4_; the extract solution was determined by UV-2450 ultraviolet spectrophotometer (Shimadzu Corp., Kyoto, Japan) at 565 nm wavelength.

Soil extracellular enzyme activity was determined by fluorescence spectrophotometry. In detail, 2.0 g of fresh soil was weighed in 125 mL buffer solution, and homogenized with magnetic stirrer for 1.5 min to make the suspension. The buffer solution was injected into the 96-well enzyme plate with the 12-channel pipette in a certain order. After 4 h culture at 25 °C, 10 μL of 1 mol L^−1^ sodium hydroxide solution was added to each hole to terminate the reaction. Then, the fluorescence data was measured with the Victor Nivo multifunctional microplate reader, PerkinElmer LLC, United States (the excitation wavelength: 365 nm, the emission wavelength: 450 nm).

Determination of soil dissolved organic matter spectral data: Ultraviolet spectral characteristics of soil samples were scanned using a UV–visible spectrophotometer (UV-1780, Shimadzu, Japan) across a wavelength range of 200–800 nm with a scanning interval of 1 nm. Three-dimensional fluorescence spectral characteristics of soil samples were scanned using a fluorescence spectrophotometer (F97 Pro, Lengguang Technology, China) equipped with a 700-V xenon lamp, across an excitation wavelength range of 200–500 nm and an emission wavelength range of 250–550 nm, with a scanning wavelength increment of 5 nm. Both excitation and emission slit bandwidths were set to 10 nm, and the scanning speed was 1,200 nm min^−1^.

UV–Vis spectral data were used to calculate the optical indices slope ratio (SR) and specific ultraviolet absorbance at 254 nm (SUVA₂₅₄), which serve as indicators for analyzing the molecular weight and aromaticity of soil dissolved organic matter, respectively. Based on the fluorescence spectral data, the fluorescence index (FI), humification index (HIX), biological index (BIX), and freshness index (β: α) were calculated to characterize the sources and transformation processes of soil dissolved organic matter.

The bacterial and fungal community compositions were analyzed using high-throughput sequencing. DNA was extracted from 0.5 g of fresh soil using the Fast® DNA Spin Kit (MP Biomedicals, Santa Ana, CA, United States) following the manufacturer’s protocol. DNA concentration and purity were verified via 1% agarose gel electrophoresis. Bacterial 16S rRNA gene V3–V4 regions were amplified using primers 515F (5′-GTGCCAGCMGCCGCGGTAA-3′) and 806R (5′-GGACT ACHVGGGTWTCTAAT-3′), while fungal ITS1 regions were amplified with primers ITS5-1737F (5′-GAAGTAAAAGTCGT AACA AGG-3′) and ITS-2-043R (5′-GCTGCGTTCTTCATCG ATGC-3′). PCR reactions (30 μL) contained 2 μL sterile ultrapure water, 15 μL Phusion® High-Fidelity PCR Master Mix, 3 μL primers (6 μM), and 10 μL template DNA (10 ng). Thermocycling conditions included initial denaturation at 98 °C for 1 min, followed by 30 cycles of 98 °C for 10 s, 50 °C for 30 s, and 72 °C for 30 s, with a final extension at 72 °C for 5 min. Amplicons were pooled, mixed with SYBR Green loading buffer, and visualized on 2% agarose gels. Sequencing libraries were prepared using the NEBNext® Ultra™ II DNA Library Prep Kit (Cat. E7645) and sequenced on the Illumina NovaSeq PE250 platform (NovaSeq, Beijing, China). For 16S rRNA, the annotation database is Silva Database,^1^ while for ITS, it is Unite Database.^2^ Index sequences were filtered using the mother pipeline. We use the DADA2 module in QIIME2 software (QIIME2 2020.2) to reduce noise to obtain the final ASVs (Amplicon Sequence Variants) and characteristic table. ASV is equivalent to clustering with 100% similarity (OTU is clustering with 97% similarity). Each ASV corresponds to a different 16S rRNA or ITS sequence. The ASV table was normalized to relative abundance for downstream statistical analyses to mitigate the effect of differing sequencing depths. All soil samples shared 34,969 bacterial ASVs and 6,223 fungal ASVs. A total of 1,117 and 51 species were detected at the level of soil bacteria and fungi, respectively. Raw data were deposited into the NCBI Sequence Read Archive (SRA) database (Accession Numbers: PRJNA1039143 [bacteria] and PRJNA1039154 [fungi]).

### 2.7 Statistical analysis

A three-way ANOVA was employed to assess the effects of straw form (F), temperature (T), and salinity (S) on bacterial and fungal communities. Data were organized in Excel 2016 (Microsoft, United States). Normality and homogeneity of variance were tested using Bartlett’s and Shapiro–Wilk tests, respectively. Significant differences in α-diversity indices (Shannon, Chao1) were evaluated via Duncan’s multiple range test in SPSS 26.0 (IBM, United States). Figures were generated using Origin 2023 (OriginLab, United States). Principal coordinate analysis (PCoA) based on Bray-Curtis distances was performed to visualize microbial community structure. Permutational multivariate analysis of variance (PERMANOVA) was then conducted using the adonis function in the R package vegan (v2.4-6) to assess the effects of straw form, temperature, and salinity on microbial communities. To validate the robustness of the PERMANOVA results, the homogeneity of multivariate dispersions was tested using the betadisper function. The Mantel tests, and partial least squares path modeling (PLS-PM) was conducted using the “vegan,” “ggplot2,” “ggcor,” and “plspm” packages in R (v4.1.2).

Microbial Co-occurrence Network Construction: ASVs detected in ≥50% of samples were retained for network analysis to balance statistical robustness against the exclusion of rare taxa, retaining 27,975 bacterial and 4,978 fungal ASVs. To account for the compositional nature of amplicon sequencing data, ASV counts were transformed using centered log-ratio (CLR) transformation prior to analysis. Spearman correlations (|*r*| > 0.95, *p* < 0.05) between ASVs were calculated using the “Hmisc” package in R. Significant correlations were imported into Gephi (v0.9.7) to visualize co-occurrence networks and compute topological parameters (e.g., modularity, centrality). Undirected networks were constructed using the “igraph” package in R.

## 3 Results

### 3.1 Alpha (α) diversity of bacterial and fungal communities in saline soils under different straw forms application

The effects of treatments on α-diversity indices of soil bacterial and fungal communities are summarized in Table 2. Three-way ANOVA results indicated that straw form significantly influenced the Shannon index of bacterial communities. Incubation temperature significantly affected all three α-diversity indices (Chao 1, Shannon, and Simpson) of bacterial communities, while soil salinity showed no significant impact. Except for the interactive effects of soil salinity × straw form on bacterial Chao 1 and Shannon indices, no other interactions significantly influenced bacterial α-diversity. Among straw treatments, no significant differences were observed in bacterial α-diversity indices, though the Shannon index under chopped straw (CW) increased by 1.7% compared to the no-straw control (CK). Bacterial α-diversity indices (Chao 1, Shannon, and Simpson) peaked at T2 (20 °C) and were lowest at T3 (30 °C). No significant differences in bacterial α-diversity were detected across soil salinity levels.

Fungal α-diversity exhibited higher sensitivity to straw form, temperature, and salinity than bacterial communities. Straw form was the dominant factor, significantly affecting all three fungal α-diversity indices. Temperature significantly influenced the Shannon index but not Chao 1 or Simpson indices, while salinity significantly altered the Simpson index. All two-way and three-way interactions among factors (straw × temperature, straw × salinity, temperature × salinity, and straw × temperature × salinity) significantly impacted fungal α-diversity. Biochar-amended soils (BW) showed the highest fungal α-diversity indices. Compared to CW, BW and granular straw (GW) significantly increased Chao 1 by 32.6 and 26.5%, Shannon by 22.9 and 18.3%, and Simpson by 8.0 and 5.7%, respectively. While CW and CK showed comparable Chao 1 and Shannon indices, CW exhibited a significantly higher Simpson index than CK. Fungal Chao 1 peaked at T3 (30 °C), surpassing T1 (10 °C) and T2 by 26.2 and 12.0%, respectively. Shannon indices at T2 and T3 were statistically similar but higher than T1, whereas Simpson indices did not differ across temperatures. Fungal α-diversity indices (Chao 1, Shannon, Simpson) were highest under moderate salinity (S2), with no significant differences between S1 (mild) and S3 (severe).

### 3.2 Composition of bacterial and fungal communities in saline soils under different straw morphologies

**Figure 1:**
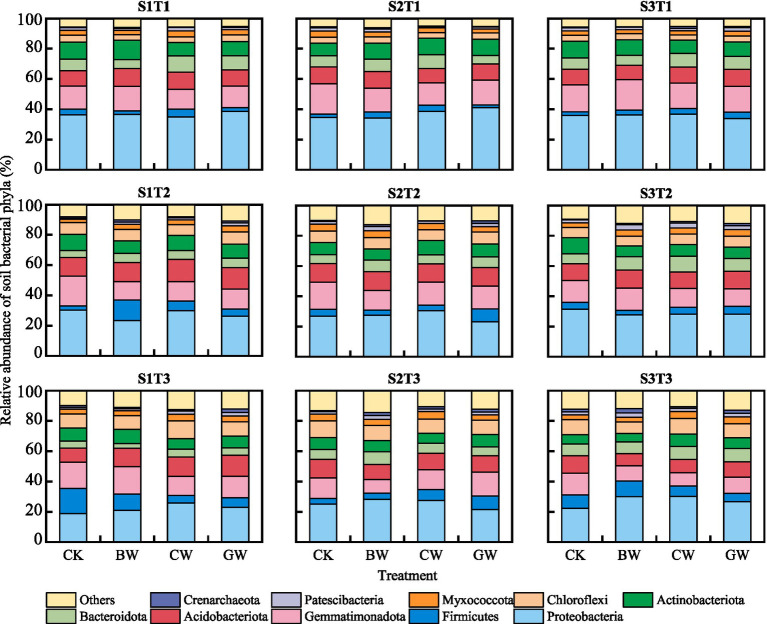
Composition of soil bacterial communities under different treatments. The data shows the top 10 taxonomic compositions of soil bacterial communities at the phylum level. “Others” represent the sum of all taxa with relative abundances lower than the top 10, or taxa that were not consistently present across all samples.

The relative abundances of dominant bacterial phyla across treatments are illustrated in Figure 1. All soils were dominated by *Proteobacteria*, *Firmicutes*, *Gemmatimonadota*, *Acidobacteriota*, *Bacteroidota*, *Actinobacteriota*, *Chloroflexi*, *Myxococcota*, *Patescibacteria*, and *Crenarchaeota*. While dominant phyla were consistent across treatments, their relative abundances varied. *Proteobacteria* was the most abundant phylum (18.9 –41.0%), followed by *Gemmatimonadota* (8.8 –20.0%) and *Acidobacteriota* (8.0 –14.8%). Straw form had no significant effect on *Proteobacteria* abundance, but temperature influenced its dominance: T1 (36.5%) > T2 (27.8%) > T3 (25.0%). *Proteobacteria* abundance was lowest under S1 salinity (27.8%), with no difference between S2 and S3. Chopped (CW) and granular straw (GW) treatments showed higher *Gemmatimonadota* abundance than biochar (BW), with T1 > T2 > T3 and S2 > S3 > S1 trends. *Acidobacteriota* abundance peaked at T2, with S1 > S2 > S3 across salinities. *Bacteroidota* abundance was consistently higher in straw-amended soils than in CK.

**Figure 2:**
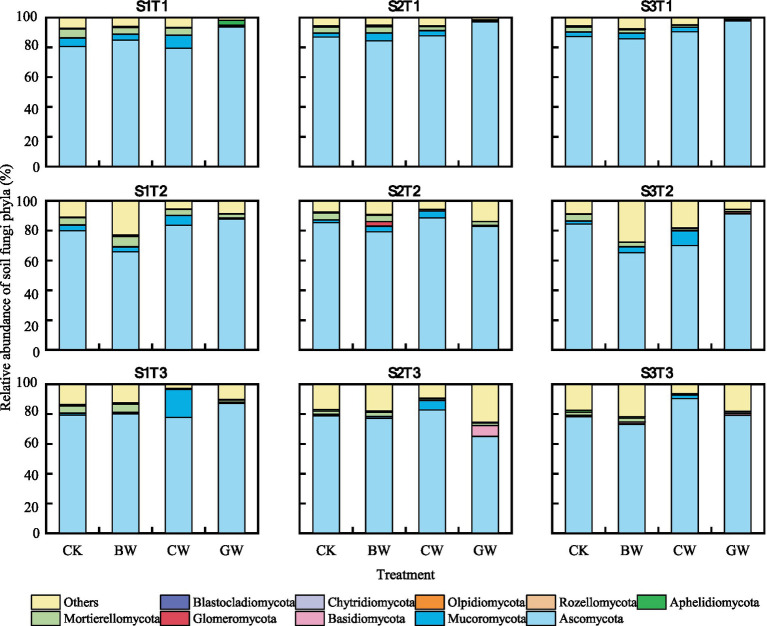
Composition of soil fungi communities under different treatments. The data shows the top 10 taxonomic compositions of soil fungi communities at the phylum level. “Others” represent the sum of all taxa with relative abundances lower than the top 10, or taxa that were not consistently present across all samples.

As shown in Figure 2, the dominant fungal phyla across treatments were similar and included *Ascomycota*, *Mucoromycota*, *Basidiomycota*, *Glomeromycota*, *Mortierellomycota*, *Aphelidiomycota*, *Rozellomycota*, *Olpidiomycota*, *Chytridiomycota*, and *Blastocladiomycota*. Among these, *Ascomycota* dominated, accounting for 65.0%–97.7% of the total fungal community. *Mucoromycota* and *Mortierellomycota* ranked second and third, contributing 0.2%–8.6% and 0.5%–5.8%, respectively. Straw application increased *Ascomycota* abundance compared to the control (CK), with the highest relative abundance observed in granular straw (GW) treatment (86.8%), exceeding chopped straw (CW) and biochar (BW) by 4.1% and 12.3%, respectively. *Ascomycota* abundance decreased with increasing temperature (T1 > T2 > T3), but showed no significant variation across soil salinity levels. For *Mucoromycota* and *Mortierellomycota*, the highest abundances were observed in CW (7.0%) and BW (3.8%) treatments, respectively. Both phyla peaked at T2 (20 °C) and under mild salinity (S1).

### 3.3 Distribution of bacterial and fungal communities in saline soil under different forms of straw application

**Figure 3:**
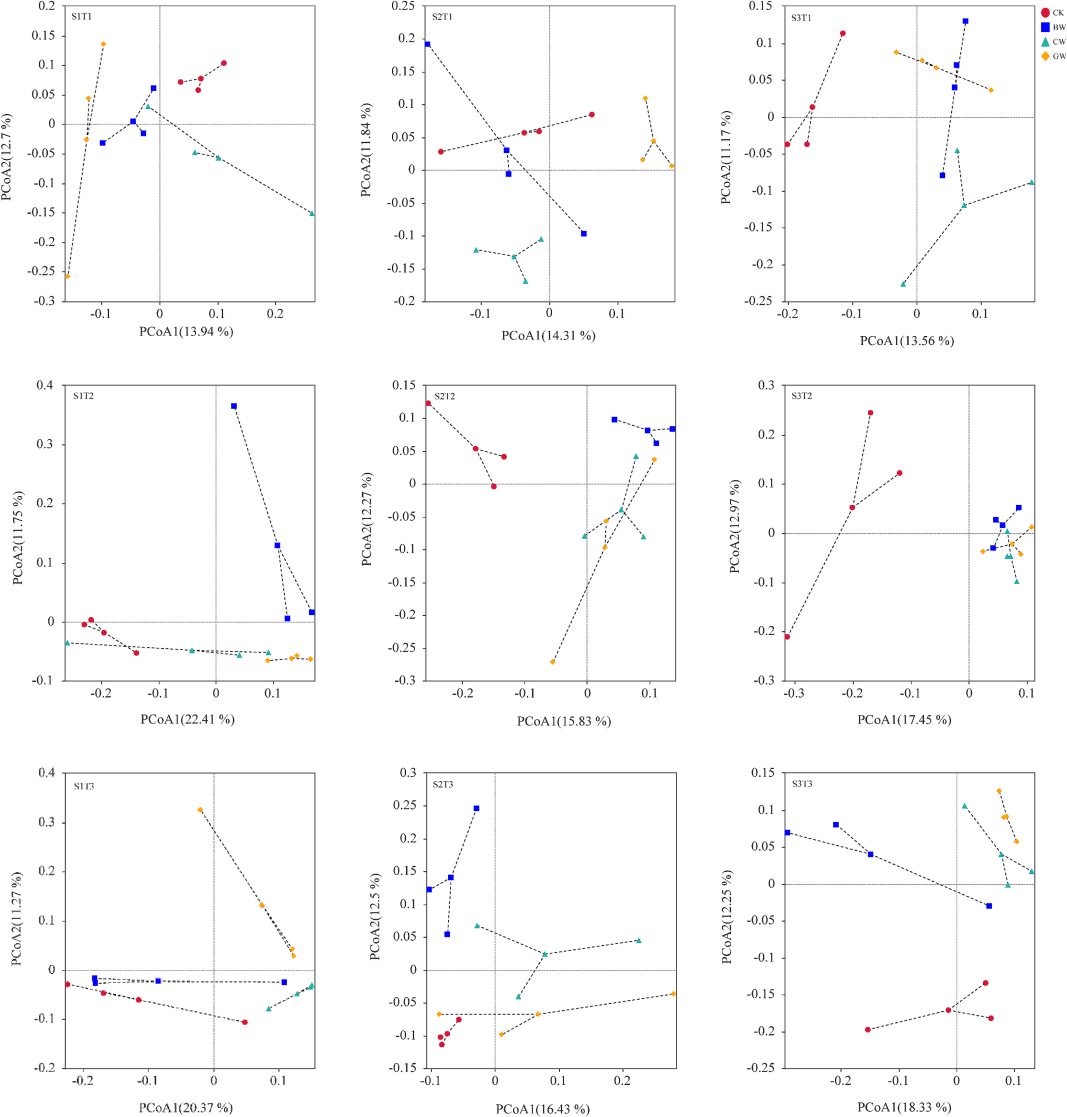
Distribution of soil bacterial communities under different treatments.

The distribution of soil bacterial communities under different treatments is shown in Figure 3. The results of Principal Coordinate Analysis (PCoA) showed that under the same environmental temperature and soil salinity, the application of different forms of straw resulted in the separation of soil bacterial communities. Among them, compared with the treatment without added straw (CK), the soil bacterial community in the straw treatment was significantly separated. At different environmental temperatures, the two principal coordinates PCoA1 and PCoA2 explain 13.56%–14.31% and 11.17%–12.70%, respectively, at T1 temperature; PCoA1 and PCoA2 at T2 temperature explain 15.83%–22.41% and 11.75%–12.97%, respectively; PCoA1 and PCoA2 at T3 temperature explain 16.43%–20.37% and 11.27%–12.50% of community dispersion, respectively. Under different soil salinity conditions, under S1 soil salinity, the two principal coordinates PCoA1 and PCoA2 explain 13.94%–22.41% and 11.27%–12.70% of the community distribution differences, respectively; PCoA1 and PCoA2 under S2 salinity explain 14.31%–16.43% and 11.84%–12.50%, respectively; Under S3 salinity, PCoA1 and PCoA2 explain 13.56%–18.33% and 11.17%–12.90% of community dispersion, respectively.

**Figure 4:**
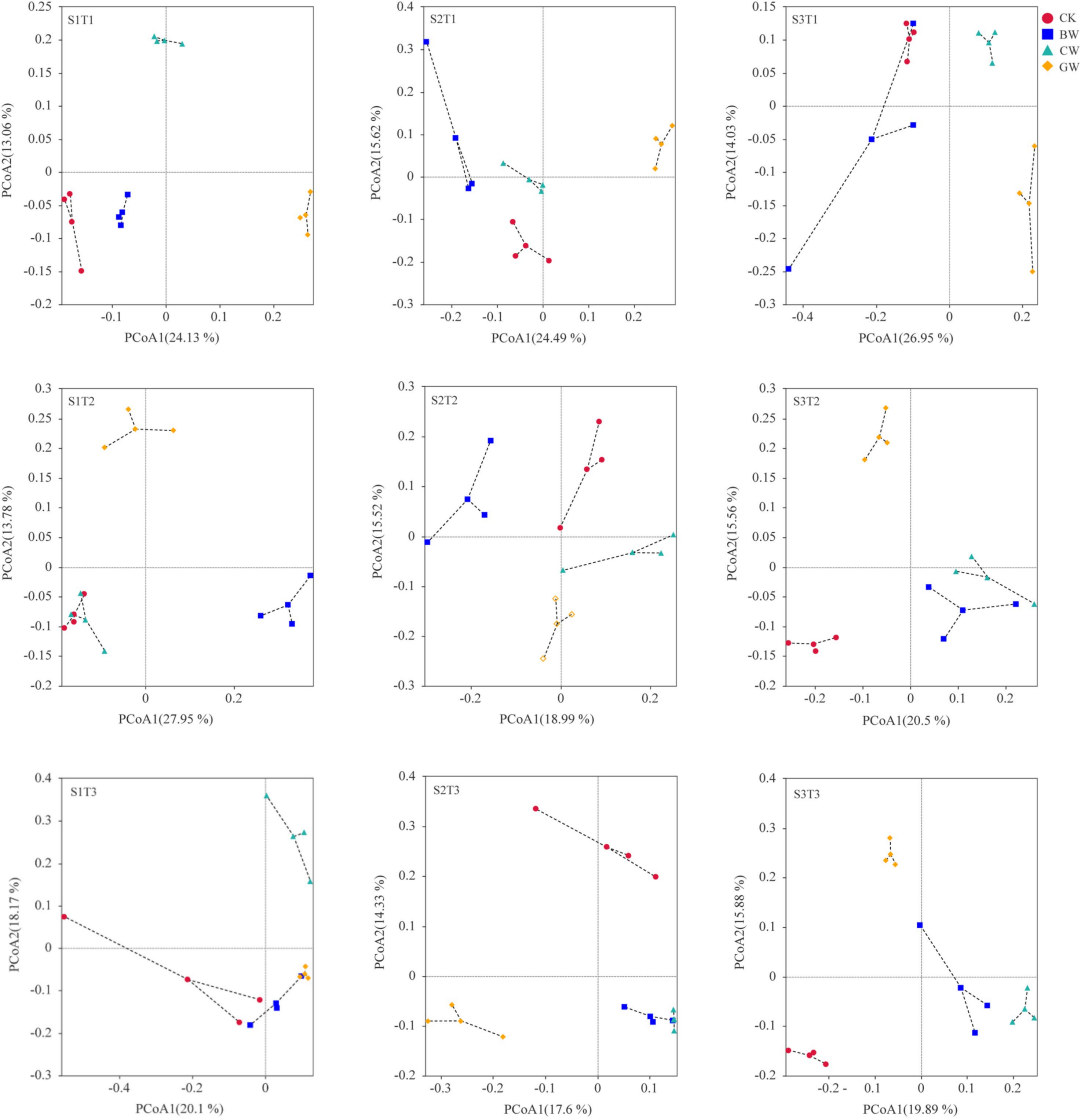
Distribution of soil fungi communities under different treatments.

As shown in Figure 4, under the same environmental temperature and soil salinity, the soil fungal communities of each treatment were significantly separated according to the different forms of straw application, and the separation effect of soil fungal communities in each treatment was more obvious than that of soil bacterial communities. The soil fungal communities treated with chopped straw (CW), biochar (BW), and granular straw (GW) were significantly separated from the CK treatment, and the soil fungal communities between the three forms of straw application were also significantly separated. Among them, the distribution of soil fungal communities in the GW treatment differed significantly from other treatments. Between different environmental temperatures, at T1 temperature, the two principal coordinates PCoA1 and PCoA2 explain 24.13%–26.95% and 13.06%–15.62% of the degree of community separation, respectively; PCoA1 and PCoA2 at T2 temperature explain 18.99%–27.95% and 13.78%–15.56%, respectively; At T3 temperature, PCoA1 and PCoA2 explain 17.60%–20.10% and 15.88%–18.17% of the community distribution differences, respectively. The PCoA results under S1 salinity for different soil salinities indicate that the first two coordinates PCoA1 and PCoA2 explain 20.10%–27.95% and 13.06%–18.17% of community dispersion, respectively; PCoA1 and PCoA2 under S2 salinity explain 17.60%–24.49% and 14.33%–15.62%, respectively; Under S3 salinity, PCoA1 and PCoA2 explain 19.89%–26.95% and 14.03%–15.88% of community segregation, respectively.

The significant differences in soil microbial community structure were tested (Table 3). The analysis of similarities (ADONIS) results showed that the differences in soil bacterial (*R*^2^ = 0.505, *p* = 0.001) and fungal communities (*R*^2^ = 0.563, *p* = 0.001) among different treatments were significant. Meanwhile, the multivariate dispersion analysis (betadisper) indicated no significant differences in the dispersion (variance) of data among groups (bacteria: *F* = 1.23, *p* > 0.05; fungi: *F* = 1.36, *p* > 0.05), confirming the robustness of the permutational multivariate analysis of variance (PERMANOVA) results. These findings suggest that straw form, environmental temperature, and soil salinity collectively drove the significant separation in the distribution of soil bacterial and fungal communities.

**Table 3:** Analysis of similarity (ADONIS) in distribution of soil bacterial and fungal communities under different treatments.

Soil microorganism	Statistic *R*^2^	*P*
Bacterial	0.505	0.001
Fungi	0.563	0.001

The *R*^2^ refers to the degree of inter group and intra group differences in soil bacterial and fungal communities under different treatments.

### 3.4 Co-occurrence network of bacterial and fungal communities in saline soil under different forms of straw application

**Figure 5:**
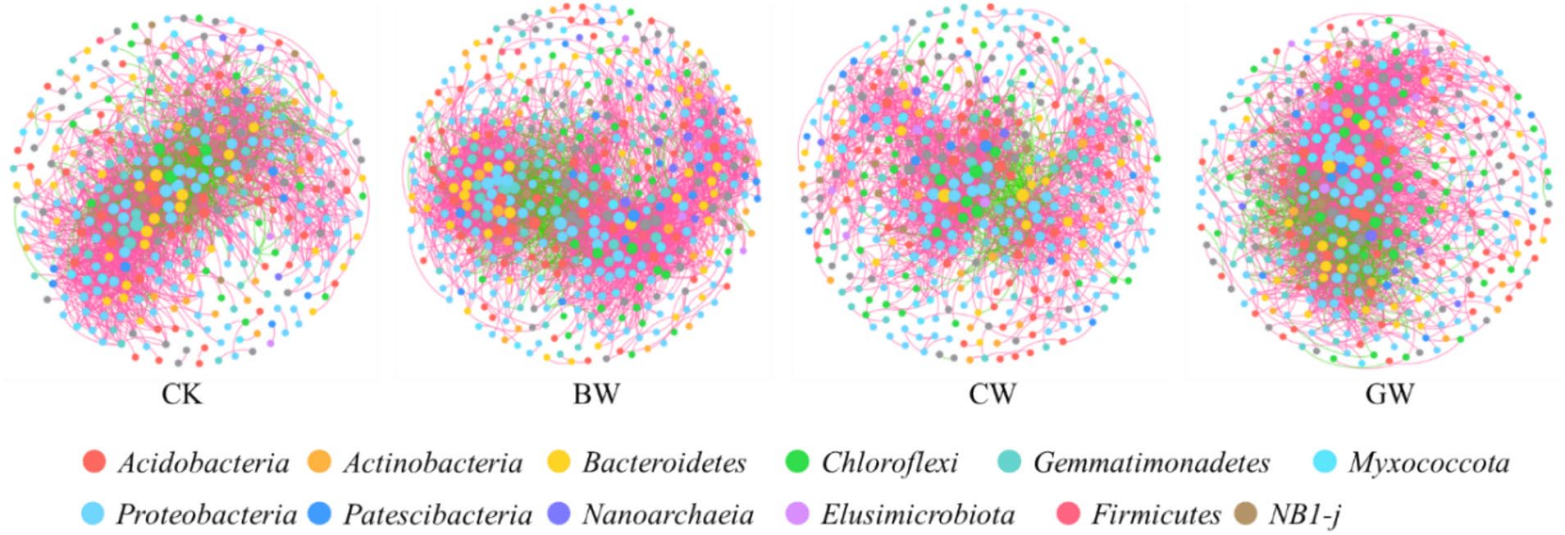
The co-occurrence network of soil bacterial communities under different forms of straw application. Only the species association with extremely significant correlation was shown (|*r*| > 0.95, *p* < 0.05), in which different nodes represent different species. The size of the node is proportional to the relative abundance of the species. Different colors represent the phylum of the ASVs. The red connection indicates a positive correlation, the blue connection indicates a negative correlation, and the number of lines indicates the intensity of the connection between the nodes. The same below.

**Figure 6:**
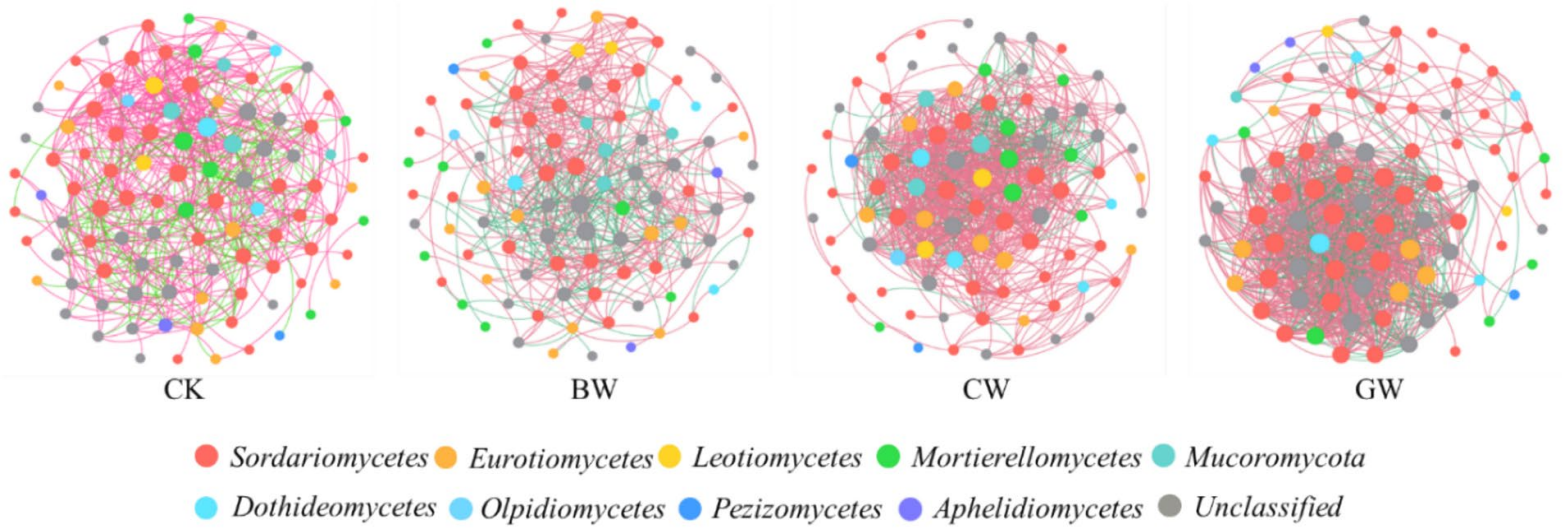
The co-occurrence network of soil fungal communities under different forms of straw application.

In order to further explore the coexistence patterns and changes in network topology of soil microbial species under different forms of straw application conditions, a co-occurrence network of soil bacterial community (Figure 5) and fungal community (Figure 6) was constructed. Compared with no straw addition (CK), the application of granular straw (GW) and biochar (BW) significantly increased the number of nodes and edges in the soil bacterial network, while there was no significant difference between the application of chopped straw (CW) and CK treatment (Table 3). Compared with CK, BW and GW treatments increased the average degree, network diameter, network density, and clustering coefficient of bacterial networks, and GW treatment had a higher modularity coefficient. Therefore, GW treatment significantly increased the complexity of soil bacterial co-occurrence networks. In addition, compared with CK, the addition of different forms of straw increased the positive correlation of bacterial networks, indicating that straw addition increased the mutualistic relationship between bacterial communities.

The core species in the microbial network were identified through centrality ranking, with higher scores indicating greater impact on the stability of the microbial network community and their important role in regulating community function. The results of the soil bacterial co-occurrence network showed that the addition of different forms of straw changed the distribution of core species in the co-occurrence network (Figure 5). The soil bacterial network treated with CK is mainly dominated by core groups such as *Proteobacteria*, *Gemmatimonas*, *Acidobacteria,* and *Bacteroidetes*. The core groups of bacterial networks treated by BW are *Proteobacteria*, *Gemmatimonadete*, *Bacteroidetes*, *Acidobacteria*, *Chloroflexi*, *Actinobacteria*. The core groups of bacterial networks under CW treatment are Proteobacteria *Gemmatimonadetes*, *Actinobacteria, Actinobacteria, Chloroflexi, Myxococcota.* The core groups of bacterial networks under GW treatment are *Proteobacteria, Gemmatimonadetes, Chloroflexi, Acidobacteria, Bacteroidetes, Myxococcota.*

For the co-occurrence network of soil fungi (Figure 6). Compared with CK treatment, CW and GW treatment significantly increased the number of fungal network edges, average degree, network density, and clustering coefficient, and GW treatment had a higher network modularity coefficient. In addition, compared with CW, the proportion of negative correlation in fungal networks increased under GW treatment (Table 3). The fungal co-occurrence network under GW treatment exhibited greater complexity. Notably, the increased proportion of negative correlations observed under GW treatment may reflect intensified competitive or antagonistic interactions, as well as finer niche partitioning among fungal taxa. From an ecological perspective, such competitive relationships can contribute to community stability by preventing any single species from dominating in response to environmental fluctuations. Thus, while the increased negative correlations indicate more complex interactions, they may also imply enhanced community resilience. The key groups for CK treatment of fungal networks are *Ascomycot* (*Sordariomycetes*) and Mortierellomycota (*Mortierellomycotes*). The fungal network under different forms of straw addition treatments is mainly influenced by the key groups Ascomycota (*Sordariomycetes* and *Eurotiomycetes*) (Table 4).

**Table 4:** Key topological characteristics and taxonomic group association networks of microbial functional traits under different treatments.

Topological parameters	Bacteria network property				Fungi network property			
	CK	BW	CW	GW	CK	BW	CW	GW
Nodes	366	471	420	411	101	100	92	88
Edges	2,865	4,406	2,235	3,696	634	494	868	992
Average degree	15.66	18.71	10.64	17.99	12.55	9.88	18.87	22.55
Network diameter	10	12	13	13	7	7	6	7
Average path length	3.10	3.43	3.70	3.46	2.46	2.74	2.20	2.48
Network density	0.04	0.04	0.03	0.05	0.13	0.10	0.21	0.26
Average clustering coefficient	0.51	0.53	0.45	0.51	0.49	0.55	0.70	0.75
Modularity	0.88	0.72	0.70	0.90	0.70	0.61	0.44	0.71
Positive correlation (%)	78.57	89.47	90.65	82.66	73.66	67.81	78.46	64.82
Negative correlation (%)	21.43	10.53	9.35	17.34	26.34	32.19	21.54	35.18

### 3.5 The relationship between soil bacterial and fungal communities and soil properties

**Figure 7:**
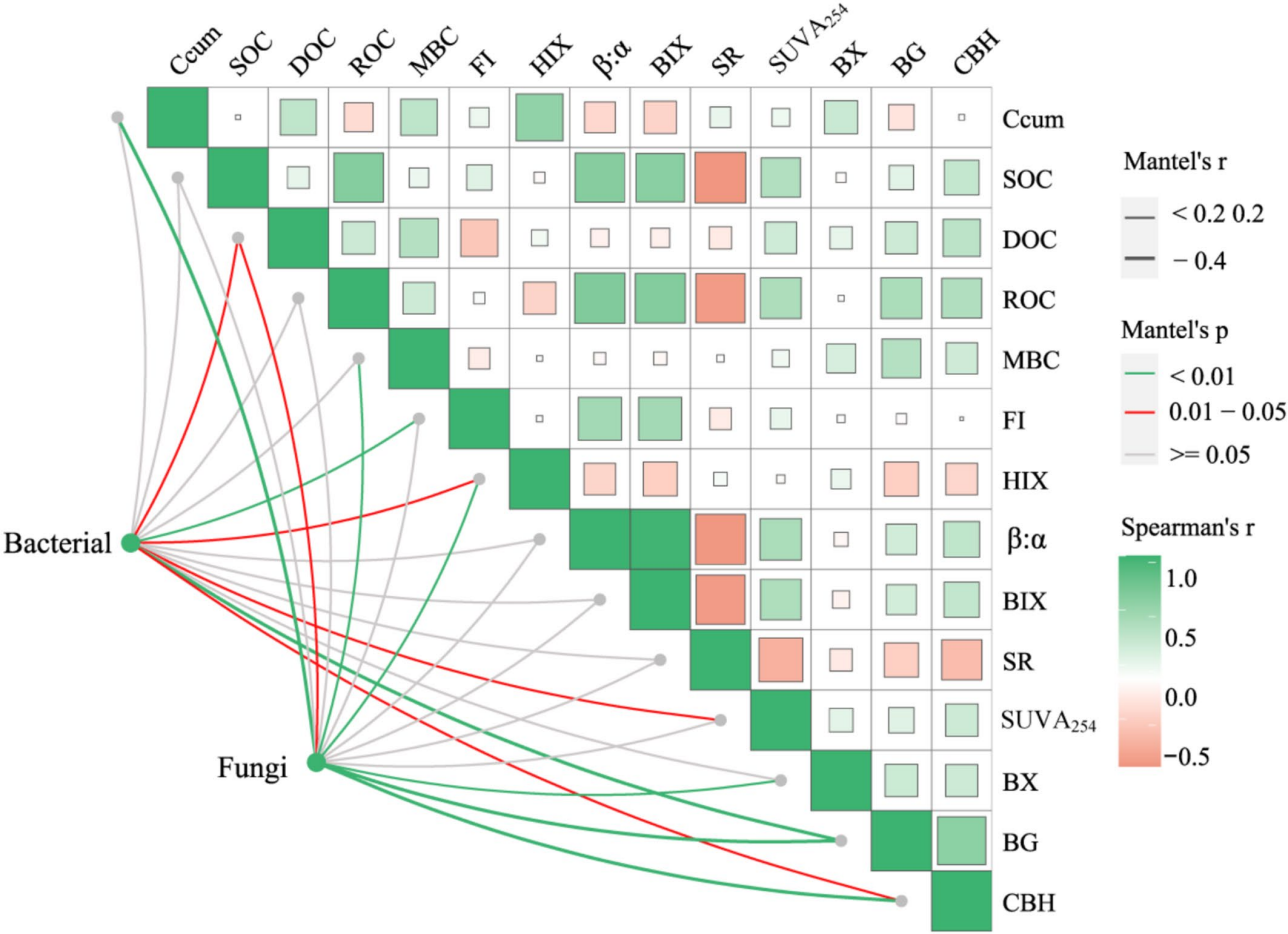
Mantel test on the relationship between soil bacterial and fungal communities and soil properties. The color gradient of the box represents Spearman’s correlation coefficient. The width of the line represents the Mantel statistic of distance correlation. The color of the line represents the significance of the correlation between soil bacteria and fungi and soil properties based on soil DNA amplicon variation sequences (ASVs) data. Ccum, Cumulative carbon mineralization; SOC, soil organic carbon; DOC, dissolved organic carbon; MBC, microbial biomass carbon; ROC, readily oxidizable organic carbon; FI, fluorescence index; HIX, humification index; β: α: freshness index; BIX, biological index; SR, slope ratio; SUVA₂₅₄, specific ultraviolet absorbance at 254 nm; BG, β-glucosidase; CBH, cellobiohydrolase; BX, β-xylosidase.

Figure 7 shows the Mantel test between soil bacterial and fungal communities and soil properties. The results indicate that the relationship between soil bacterial communities and soil organic carbon components, enzyme activity, and optical indicators of soluble organic matter is different from that between soil fungal communities and them. There is a significant positive correlation between soil bacterial community and DOC content in soil active organic carbon components. There is a significant positive correlation between soluble organic matter fluorescence index (FI), humification index (HIX), and aromaticity index (SUVA_254_). There is a significant positive correlation between BG and CBH activities in soil enzyme activity. Compared with soil bacterial communities, the relationship between soil fungal communities and soil physicochemical properties is closer, with a highly significant positive correlation between soil cumulative carbon mineralization (Ccum) and soil fungi. The soil fungal community is significantly positively correlated with soil DOC, MBC content, and HIX. There is a highly significant positive correlation with soil BX, BG, and CBH.

**Figure 8:**
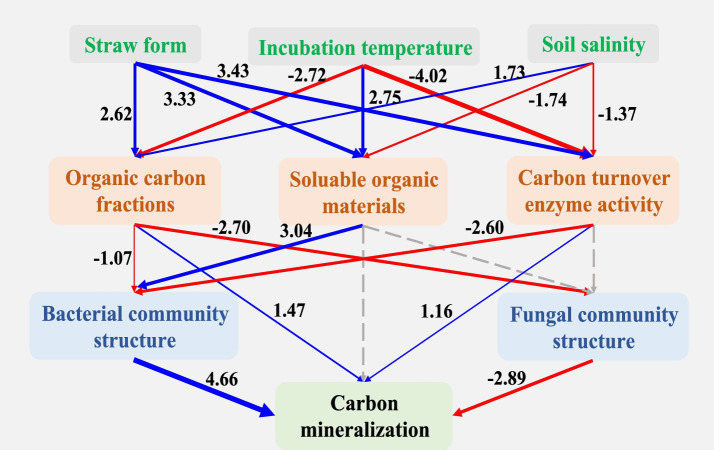
The PLS-PM analysis between soil microorganisms and soil properties. The width of the connecting arrows represents the strength of the relationship between the two factors, with the numbers indicating the standardized path coefficients. Dashed lines indicate no significant effect (*p* > 0.05), while blue and red lines represent positive and negative effects, respectively, (*p* < 0.05). The goodness of fit (GoF) statistic is utilized to evaluate the model, with a GoF value of 0.68.

The Partial Least Squares Path Model (PLS-PM) suggested the complex associations between straw form, environmental temperature and soil salinity, organic carbon components, soluble organic matter, extracellular enzymes related to carbon turnover, soil bacterial community structure, and soil fungal community structure and organic carbon mineralization (Figure 8). The form of straw showed a significant positive association with organic carbon components, soluble organic matter, and extracellular enzyme activity related to carbon turnover. Negative associations were observed between cultivation temperature, organic carbon components, and extracellular enzymes related to carbon turnover. The soil salinity level exhibited a significant negative association with soluble organic matter and extracellular enzymes related to carbon turnover. Both organic carbon components and extracellular enzyme activities related to carbon turnover were positively associated with organic carbon mineralization. The structure of soil bacterial and fungal communities showed significant associations with organic carbon mineralization.

## 4 Discussion

### 4.1 Impact of straw form on microbial community structure in saline-alkali soil

Soil microorganisms are an important component of agricultural ecosystems, serving as key control factors for soil nutrient cycling and energy conversion, and playing a crucial role in soil organic carbon turnover (Pan et al., 2025). The quantity, species diversity, and community structure of soil microorganisms are highly sensitive to environmental changes, such as soil carbon pool levels, environmental temperature, and soil salinization levels, all of which can affect the community structure of soil microorganisms (Andronov et al., 2012; Li et al., 2023; Zhang et al., 2022). Soil microorganisms adjust their ecological niche functions based on changes in these environmental conditions to meet normal physiological metabolism (Yang et al., 2025).

Returning straw to the field is a commonly used agricultural management measure to increase the soil organic carbon pool. The abundant carbon, nitrogen, and various nutrients in straw provide material and energy sources for the proliferation and metabolism of soil microorganisms. Numerous studies have shown that returning straw to the field can increase soil microbial diversity and improve microbial community structure (Cong et al., 2020b; Cui et al., 2025; Liu et al., 2022; Zeng et al., 2025). This study found that compared to environmental temperature and soil salinity, straw form is the main factor affecting soil microbial community structure. In addition, the changes in soil bacterial alpha diversity treated with different forms of straw addition were relatively small, while the addition of different forms of straw had a significant impact on soil fungal alpha diversity, and the changes in soil fungal community alpha diversity and SOC content showed a synergistic effect.

In this study, the dominant phyla of bacteria in soil treated with various forms of straw were similar, but their relative abundance varied in different treatments. This variation in relative abundance, despite compositional similarity, suggests that straw morphology primarily influences bacterial community structure by modulating resource availability rather than by selecting for distinct phyla. Chen et al. (2022) found that after returning straw to the field, *Proteobacteria*, *Firmicutes*, *Bacteroidetes*, *Actinobacteria*, and *Chloroflexia* were the soil bacterial populations with relatively high abundance in all treatments. Studies have shown that these bacterial communities are key groups involved in the decomposition and mineralization of straw in soil, and play an important role in carbon cycling (Zhang et al., 2024). The relative abundance of *Bacteroidetes* in the treatment without straw addition was lower than that in the treatment with straw addition. This is because in the “nutritional niche” model, *Bacteroidetes* belongs to the “eutrophic microbial community,” and the higher the organic carbon content in the environment, the richer the community composition (Iqbal et al., 2009). There are also studies indicating that most soil bacterial populations do not change regularly with the application of straw, and may even decrease the relative abundance of “oligotrophic bacteria” such as *Acidobacteria* (Li et al., 2020). Therefore, there may be strong competition among bacterial species that decompose organic residues such as straw, which also promotes the succession of bacterial communities, which often depends on the organic carbon and active organic carbon content in the soil. Studies have shown that soil organic carbon and active organic carbon content are important factors affecting the variability of soil bacterial communities (Song et al., 2022). This viewpoint further suggests that the differences in soil bacterial communities under different treatments may be due to the addition of different forms of straw. In the correlation analysis of this study, the Shannon and Simpson indices of soil bacteria were significantly correlated with SOC content; The significant correlation between soil bacterial community and DOC in Mantel test also confirms this viewpoint. The study by Liu et al. (2025) also suggests that the structure of soil bacterial communities depends on the content of active organic carbon and biochemical characteristics in their living environment.

In contrast to soil bacteria, the response of soil fungi to straw form was more pronounced. Among different forms of straw treatment, the soil fungal alpha diversity index was highest when using biochar. It can be seen that the application of biochar increases the number of soil fungal communities. This may be due to the strong reproductive ability of soil fungi in slightly alkaline environments, and according to our research, among the three forms of straw, only the pH of biochar is slightly alkaline. The study by Adekiya et al. (2025) also confirmed this viewpoint. In addition, this may also be due to the promoting effect of biochar on soil aggregate formation, which improves the living environment of soil fungi (Wang F. et al., 2025). This study found that compared to cutting straw, the application of granular straw significantly increased the soil fungal alpha diversity index. This should be due to the expansion of granular straw in soil, which can improve soil structure and increase aggregate content (Fan et al., 2023). In addition, as expected, the addition of different forms of wheat straw is related to changes in soil microbial community composition and alters their relative abundance and community distribution. After applying different forms of straw, there were significant changes in the community levels of soil fungi, indicating that fungal communities may be more sensitive than bacteria to the physical structure of organic amendments. Cong et al. (2020b) also obtained similar results in their study. This study found that the dominant phyla of fungi in soil treated with various forms of straw were similar, but their relative abundance varied in different treatments. The relative abundance of *Ascomycota* in soil treated with granular straw was higher than that in soil treated with chopped straw and biochar. The higher abundance of *Ascomycota* in the granular straw treatment aligns with its classification as a eutrophic phylum. This is consistent with our earlier report that granular straw application significantly increases soil SOC content (Fan et al., 2024), thereby creating a favorable niche for eutrophic communities like *Ascomycota*. It is important to note, however, that increased fungal dominance does not necessarily indicate enhanced carbon use efficiency (CUE) and may instead reflect higher respiratory costs associated with enzyme production (Zhou et al., 2025; Wu et al., 2022; Soares and Rousk, 2019). However, the relative abundance of *Ascomycota* in soil treated with biochar is lower, which may be due to the difficulty in utilizing its polycyclic aromatic carbon structure. In contrast to soil bacteria, most soil fungi are capable of both decomposing labile components in straw, such as polysaccharides and fats, and secreting extracellular enzymes to efficiently break down recalcitrant compounds like cellulose and lignin (Cong et al., 2020a; Liu et al., 2022; Yang et al., 2023). BX, CBH, and BG can degrade cellulose and hemicellulose in straw into glucose. The Mantel test results also showed a significant correlation between soil fungal communities and the activity of three soil extracellular enzymes. Ge et al. (2021) also showed that soil fungi can synthesize extracellular enzymes to degrade complex carbon structures. This should be due to the application of straw promoting the growth of soil fungi, which can produce various extracellular depolymerases to explore nutrients and redistribute energy through their filaments. Thus, while GW enhanced fungal abundance and network complexity, this likely reflects a shift in microbial decomposition strategy—favoring the breakdown of recalcitrant carbon—rather than a direct improvement in carbon retention efficiency. Future studies incorporating direct measurements of microbial CUE and growth yield are needed to confirm the net carbon balance implications of this community shift.

In addition, we found that the application of granular straw significantly stimulated the complexity of soil bacterial and fungal community co-occurrence networks, promoting connectivity within and between co-occurrence network modules. The addition of granular straw increased the proportion of negative correlations among microbial species. In co-occurrence network analysis, positive interactions typically suggest shared ecological preferences or cooperative relationships, whereas negative interactions often indicate competitive exclusion or antagonism. A higher proportion of negative correlations suggests that granular straw fosters intensified competitive or antagonistic interactions within the microbial community. This enhanced competitive network can buffer against external environmental disturbances, as competitive relationships may stabilize the community by preventing any single species from dominating in response to fluctuating conditions. Consequently, this maintains mutual constraints among microbial taxa and improves overall network stability. The study by Zhou et al. (2020) also suggests that different types of microorganisms in soil microbial networks may maintain their balance through negative correlations, thereby improving the health and stability of soil ecosystems. In summary, we believe that different forms of straw returning to the field have changed the quantity and structure of soil bacterial and fungal communities, with the most significant differences in the distribution of granular straw communities, closer connections between groups, and better microbial cooperation.

### 4.2 Impact of environmental temperature on microbial community structure in saline-alkali soil

The mechanism by which environmental temperature regulates soil microbial ecological functions involves multiple aspects, including organic matter decomposition, extracellular enzyme activity, and nutrient cycling (Zhang et al., 2022). The adaptability and competitiveness of soil bacteria and fungi vary under different environmental temperatures, and their metabolic pathways are closely related to temperature changes. Studies have shown that temperatures that are too high or too low can inhibit the activity of soil microorganisms (Nicolardot et al., 1994). For soil bacteria, this study found that at different temperatures, compared with T1 (10 °C) and T3 (30 °C), the soil bacterial community alpha diversity index (Chao 1, Shannon, Simpson) was highest at T2 (20 °C), suggesting that moderate temperatures optimize niche partitioning and support more diverse bacterial assemblages. This is consistent with the findings of Frossard et al. (2021). Furthermore, studies have shown that temperature changes can alter the quantity of soil microorganisms, but not the species composition of their communities (Hopkins et al., 2014), reinforcing the idea that temperature acts primarily as a regulator of microbial activity and abundance, rather than a filter for community composition. We observed that the composition of dominant bacterial phyla remained similar across different temperatures, while their relative abundance shifted. Specifically, both *Proteobacteria* and *Zygomycota* exhibited a decreasing trend with increasing temperature (T1 > T2 > T3), which may reflect differential thermal tolerance or competitive dynamics under warming conditions. This trend is similar to the results of He et al. (2023). However, some studies have reported that higher temperatures increase the relative abundance of *Proteobacteria*, a discrepancy that likely stems from differences in background soil properties or climatic contexts, highlighting the context-dependency of microbial thermal responses (Arunrat et al., 2023). Additionally, this study found that the relative abundance of Acidobacteria peaked at 20 °C, implying an optimal temperature range for this phylum and potential vulnerability to both cooling and warming, which is consistent with the findings of Foesel et al. (2014).

In contrast, soil fungal communities exhibited different response patterns. We found that only the Chao 1 index of fungal alpha diversity was highest at T3 (30 °C), indicating the highest total number of species at this temperature, while other diversity indices showed no significant differences. This suggests that soil fungi are less sensitive to temperature changes than bacteria. Newsham et al. (2022) also reported that soil fungal diversity increases with rising temperature. When temperature increases, microbial metabolic activity strengthens, proliferation accelerates, and the decomposition of organic matter speeds up, leading to increased CO₂ emissions (Qi et al., 2025; Xiong et al., 2014; Zhang Z. et al., 2021). However, some studies indicate that increasing temperature may reduce fungal abundance, which could be related to specific study areas and soil types (Feng and Simpson, 2009). At the phylum level, we observed that the relative abundance of *Ascomycota* decreased with increasing temperature. Xiong et al. (2014) also showed that while specific members of *Basidiomycota* increased in abundance with warming, *Ascomycota* exhibited a range of responses.

Across these divergent responses, both bacterial and fungal communities demonstrated that temperature effects on microbial composition may vary across different climate zones and soil types. The impact of temperature on soil microbial communities is complex and context-dependent. Therefore, in future research, the role of environmental temperature in the ecosystem functional mechanisms of soil microorganisms should be further investigated for specific microbial groups, in order to better address climate change and maintain ecosystem balance.

### 4.3 Impact of soil salinity level on microbial community structure in saline-alkali soil

The change in soil salinity leads to changes in the osmotic pressure and nutrient conditions of soil water, which will inevitably alter the structure of soil microbial communities (Haj-Amor et al., 2022). For soil bacteria, the results of this study indicate that their alpha diversity is insensitive to changes in soil salinity. The relative abundance of specific bacterial phyla, however, showed distinct responses: *Proteobacteria* and *Bacteroidetes* exhibited their lowest relative abundance in low-salinity soils, while *Acidobacteria* had its lowest abundance in high-salinity soils. This pattern suggests that *Proteobacteria* and *Bacteroidetes* possess relatively high salt tolerance, whereas *Acidobacteria* is more sensitive to increasing salinity. These differential responses underscore the role of salinity as an environmental filter that reshapes microbial community structure by favoring osmotolerant taxa. These findings are consistent with Wang C. et al. (2025) regarding the response of soil microbial diversity to salinity gradients.

In contrast, soil fungal communities exhibited significant changes in alpha diversity across different salinity levels, suggesting that fungi may have narrower salinity tolerance ranges or be more constrained by osmotic stress than bacteria. This is similar to the findings of Wei et al. (2024). Specifically, fungal alpha diversity was significantly higher under moderate salinization than in low- or high-salinity soils. This may be attributed to an appropriate increase in salt content improving the nutritional conditions for certain salt-tolerant microorganisms, thereby enhancing their abundance and diversity, while excessive salinity inhibits nutrient absorption and physiological metabolism (Boyrahmadi and Raiesi, 2018). Yang et al. (2020) also reported that high salinity reduces soil fungal diversity. At the phylum level, the relative abundance of *Ascomycota* was highest in low-salinity soils, further supporting this view.

Despite these differences, some studies have reported that high salt stress did not alter the presence or abundance of dominant phyla such as *Proteobacteria*, *Acidobacteria*, and *Ascomycota* (Xia et al., 2023). This discrepancy may be due to variations in salt types and soil moisture conditions, warranting further investigation. Furthermore, correlation analyses between soil microbial (bacterial and fungal) alpha diversity and community composition with soil carbon fractions and enzyme activities, along with Mantel test results, indicate that soil microorganisms secrete relevant extracellular enzymes in response to soil salinity conditions, thereby promoting soil carbon pool turnover and nutrient cycling.

## 5 Conclusion

This study elucidated the interactive effects of straw form, ambient temperature, and soil salinity on soil microbial communities and C cycling processes. The results identified straw form as the dominant driver regulating both bacterial and fungal alpha-diversity. Specifically, BW markedly enhanced fungal diversity, followed by GW, whereas CW yielded the lowest values. An ambient temperature of 20 °C was associated with the highest microbial diversity, and moderate salinization significantly increased fungal alpha-diversity. Straw addition, particularly the GW treatment, significantly increased the relative abundance of the dominant fungal phylum, *Ascomycota* (>65%), an effect further amplified by decreasing temperatures (T1 > T2 > T3). Straw form also significantly shaped microbial community structure. The GW treatment induced distinct separation of the fungal community from other treatments and enhanced the complexity of bacterial-fungal co-occurrence networks, indicating more complex ecological interactions between these microbial groups. Critically, analysis of key environmental drivers revealed distinct regulatory pathways: Straw form positively influenced organic C fractions, DOC and C-hydrolyzing enzyme activities (e.g., BG, CBH). Temperature exhibited negative correlations with these organic C fractions and enzyme activities. Conversely, salinity significantly suppressed DOC and C-cycling-related enzyme activities. Collectively, these findings provide insights into the mechanisms governing microbially mediated C cycling during straw utilization and offer a theoretical basis for developing soil carbon sequestration strategies in agricultural systems.

## Data Availability

The original contributions presented in the study are included in the article/supplementary material, further inquiries can be directed to the corresponding author.

## Footnotes

1
https://www.arbsilva.de/

2
https://unite.ut.ee/
